# Right Coronary Artery STEMI following Blunt Thoracic Trauma

**DOI:** 10.1155/2021/5598524

**Published:** 2021-08-17

**Authors:** Saad Sikanderkhel, Jon-Austin Ash, Kimberly O'Dell, Jacob Mok, Harish Manyam, John Golding

**Affiliations:** ^1^Department of Cardiovascular Medicine, University of Tennessee College of Medicine Chattanooga, USA; ^2^Department of Internal Medicine, University of Tennessee College of Medicine Chattanooga, USA; ^3^Heart and Lung Institute, Erlanger Baroness Hospital, Chattanooga, Tennessee, USA

## Abstract

We present a case report of an otherwise healthy 37-year-old male without pertinent cardiac history or risk factors, who presented with cardiac trauma after a motor vehicle collision (MVC). Initial workup including electrocardiogram (ECG), transthoracic echocardiogram (TTE), and uptrending serial troponins warranted coronary angiography, during which occlusive thrombi were discovered in the proximal right coronary artery (pRCA), the right posterolateral vessel (rPL), and the right posterior descending artery (PDA). Subsequently, the patient underwent percutaneous coronary intervention of the RCA and PDA with aspiration thrombectomy. He was then initiated on dual antiplatelet therapy (DAPT) and recovered appropriately. This case is presented with the goal of enlightening the academic community of a rare complication while presenting a judicious approach to management in an attempt to decrease the occurrence of “near misses” in the future.

## 1. Introduction

Blunt thoracic trauma commonly leads to blunt cardiac injury (BCI), i.e., injury of the myocardium and large vessels. BCI is reported to occur in as many as 20% of all blunt thoracic trauma cases [1]. However, amongst those cases, acute coronary artery occlusion is not common, especially occlusion of the RCA. We present the case of a young man without cardiac history or pertinent risk factors, who was found to have an acute myocardial infarction (AMI) in the setting of an RCA thrombosis secondary to blunt thoracic trauma via MVC.

## 2. Case Presentation

A 37-year-old male presented to the emergency department (ED) due to trauma sustained as the unrestrained driver in an MVC. He was without loss of consciousness, remained hemodynamically stable, but was combative with evidence of head trauma. He was sedated and intubated at the scene due to incipient airway compromise and was then transferred to the hospital by air ambulance.

**Figure 1:**
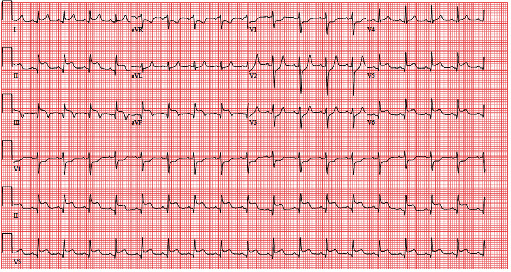
Inferolateral STEMI. ECG in the ED showing sinus tachycardia and ST-segment elevation in the inferolateral leads.

In the ED, the patient was noted to be normotensive and tachycardic while sedated on ventilator support. Physical exam was notable for multiple areas of ecchymosis and lacerations. Workup revealed multiple fractures, a small right pneumothorax, and pelvic hematoma. ECG was concerning for AMI with ST elevations in the inferior and lateral leads (Figure 1). A bedside TTE demonstrated normal left ventricular ejection fraction, mild inferolateral hypokinesis, and absence of tamponade physiology. His initial Troponin-I was 0.56 ng/ml.

**Figure 2:**
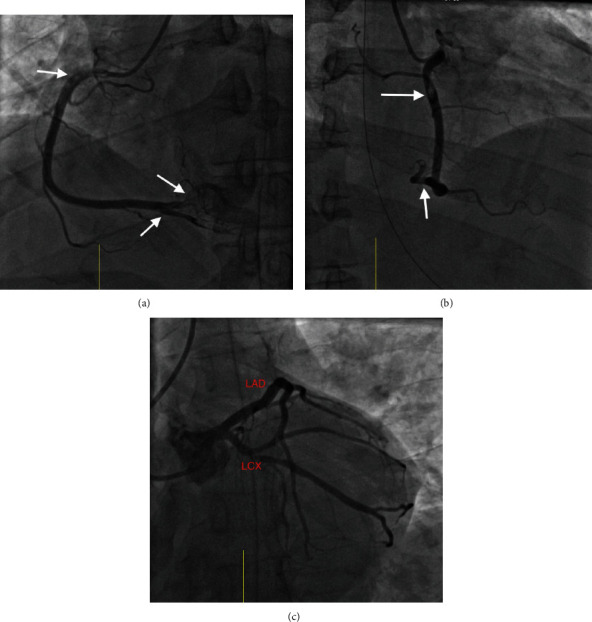
Coronary angiography prior to intervention. (a) pRCA, rPL, and PDA thrombus: coronary angiography (LAO view) showing thrombus in the proximal right coronary artery (pRCA), the right posterolateral (rPL), and the right posterior descending (PDA) branches (white arrows). (b) pRCA and rPL thrombus: coronary angiography (RAO view) showing thrombi in the pRCA and rPL branches (white arrows). (c) Normal left main, LAD, and LCX: coronary angiography (LAO cranial view) showing angiographically normal left main, left anterior descending (LAD), and left circumflex (LCX) arteries.

**Figure 3:**
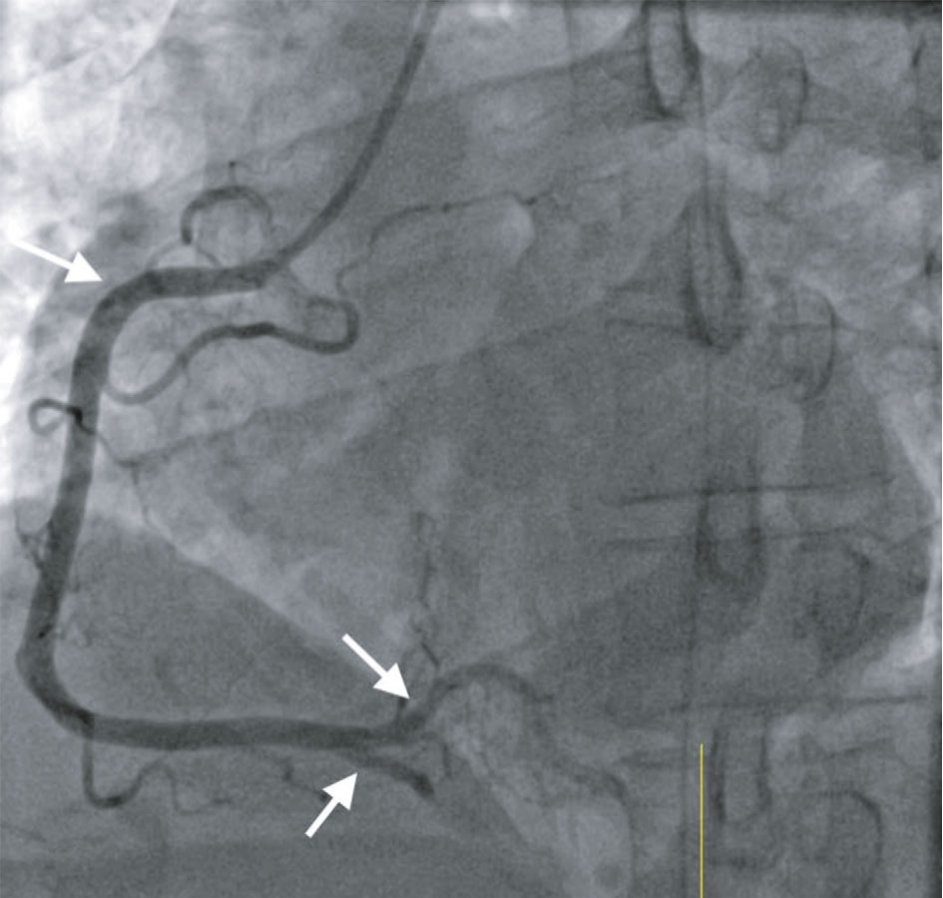
Coronary angiography after intervention. pRCA and rPL s/p aspiration thrombectomy: coronary angiography (LAO view) showing complete resolution of thrombi in the pRCA and rPL branches after aspiration thrombectomy (white arrows). However, the PDA remained occluded with microthrombi as a consequence of intervention in the proximal RCA (white arrow).

The patient was given aspirin 325 mg and subsequently observed without scheduled antiplatelet therapy due to incipient hemodynamic compromise in the setting of a hematoma and extensive trauma. In the interim, serial Troponin-I levels continued to rise sharply, peaking at 34.24 ng/ml, necessitating urgent coronary angiography via the right radial artery access, which revealed thrombosis in the pRCA, the rPL, and the distal PDA (Figure 2), at which point the patient underwent successful aspiration thrombectomy with the Pronto aspiration catheter system (Teleflex Medical OEM, Gurnee, IL, USA) without further evidence of underlying plaque or dissection. The distal PDA remained occluded in the aftermath of pRCA intervention, and attempts at aspiration thrombectomy were precluded by the small diameter of the vessel (<2 mm) (Figure 3). After the procedure, a glycoprotein IIb/IIa inhibitor infusion was administered for 12 hours. Intravenous Cangrelor, which had been initiated during the procedure, was changed to clopidogrel once additional orthopedic procedures were no longer anticipated. The patient recovered well after the procedure and was discharged home in a stable condition. He was prescribed DAPT for at least 12 months along with a high-intensity statin and beta-blocker.

## 3. Discussion

With extensive thoracic trauma, as in our case, a broader differential diagnosis needs to be considered to include cardiac contusion, large vessel dissection, external compression of the coronary artery, and stress-induced cardiomyopathy. These, along with valvular malfunction in the setting of a traumatic avulsion, pericardial effusion, and arrhythmias are known to be the most common cardiac complications resulting from blunt thoracic trauma [2]. AMI secondary to blunt thoracic trauma is rare, as direct injury to the coronary vessels is uncommon [3], occurring in only 3% of all BCIs [1]. However, this does occur with a proposed etiology due to the superficial anatomic location of the coronaries making the vessels susceptible to thrombosis formation in the setting of a traumatic event [4, 5]. Furthermore, since the left anterior descending artery is the most anterior vessel and hence in the most vulnerable position, it is also the most commonly injured [4, 6–13].

This case report of AMI with occlusion of the pRCA, rPL, and PDA represents an exceedingly rare complication of blunt thoracic trauma. Our patient had no cardiac history or risk factors for atherosclerotic coronary vascular disease, and no evidence of underlying coronary artery disease was found with angiography either. This suggests that blunt trauma-induced thrombosis was the most likely cause of the AMI. Fortunately, a complete cardiac workup upon presentation and early involvement of a cardiology team led to prompt diagnosis and treatment, which afforded the patient an excellent outcome. However, such is not always the case.

When a patient presents with blunt thoracic trauma, BCIs are often masked by precordial pain due to trauma to that area, making the diagnosis less clear [14]. For generalized cardiac complications, a workup including ECG and cardiac biomarkers is a must, as illustrated via a meta-analysis directly correlating abnormal values of such with patients requiring cardiac treatment [15]. Specifically for AMI as in our case, serial ECGs afforded the most useful diagnostic information, in addition to wall motion abnormalities noted on TTE, as well as elevated cardiac biomarkers [16]. Cardiac MRI with contrast and cardiac CT is also beneficial as has been shown useful in differentiating between cardiac contusion and acute MI [17, 18].

Although rare, providers should always maintain a high index of suspicion for BCI following blunt thoracic trauma. Workup should include, at a minimum, ECG, echocardiography, and trending of cardiac biomarkers to allow for early identification of AMI, i.e., need for coronary intervention, which is critical for improved outcomes [19–21].

## Data Availability

Data are available upon request.
